# Salvianolic acid B decreases oxidative stress and alleviates the tumor-promoting effects of arecoline in oral cancer

**DOI:** 10.1016/j.crphar.2025.100241

**Published:** 2025-11-29

**Authors:** Hsuan-Yin Tung, Yi-Ling Ye, Chi-Maw Lin, Li-Shian Shi

**Affiliations:** a Graduate Institute of Life Sciences, National Defense Medical Center, No. 161, Sec. 6, Minquan E. Rd., Neihu Dist., Taipei City, 114201, Taiwan; b Department of Biotechnology, National Formosa University, No. 64, Wunhua Rd, Huwei Township, Yunlin County, 63201, Taiwan; c Department of Otolaryngology, National Taiwan University Hospital, Yun-Lin Branch, No.579, Sec.2, YunLin Rd., Yunlin County, Douliou City, 640, Taiwan; d Graduate Institute of Clinical Medicine, College of Medicine, National Taiwan University, Taipei, Taiwan

**Keywords:** Arecoline, Salvianolic acid B, Oral cancer, Oxidative stress, Fibrosis, RNA sequencing

## Abstract

Arecoline, which is a primary alkaloid in areca nuts, contributes in key ways to the development of oral submucous fibrosis and the subsequent oral cancer through the induction of oxidative stress, promotion of fibrosis, and activation of oncogenic signaling. Salvianolic acid B (SAB) is the most abundant water-soluble phenolic compound found in *Salvia miltiorrhiza* Bunge. SAB appears to have the potential to mitigate the effects of arecoline. However, the interaction between SAB and arecoline in oral cancer has been less frequently discussed. Therefore, we conducted this study in which SCC-4 tongue cancer cells were treated with arecoline alone or in combination with SAB. The effects on collagen contraction, cell migration, reactive oxygen species (ROS) production, and transcriptomic alterations were assessed. Arecoline increased collagen contraction, ROS accumulation, and the activation of tumor-promoting pathways, including TGF-β/Smad, EGFR, MAPK, and ferroptosis. In contrast, SAB effectively decreased collagen contraction, reduced cell migration, and attenuated oxidative stress in a dose-dependent manner. Moreover, in the presence of arecoline, SAB supplementation reversed fibrosis-related processes, modulated metabolic activity, and enhanced DNA repair mechanisms, thereby counteracting arecoline-induced oncogenic effects. Therefore, SAB, through its ability to reduce oxidative stress, fibrosis, and metabolic dysregulation, is a promising therapeutic candidate for mitigating arecoline-induced tumor progression. Our study offers novel insights into the role of SAB in protecting against the pathophysiology of oral cancer and highlights its potential as a natural compound for the prevention and treatment of this disease.

## Highlights

• SAB mitigates the pro-fibrotic effect of arecoline on collagen contraction.
• SAB significantly suppresses SCC-4 cell migration in a dose-dependent manner.
• SAB significantly reduces arecoline-induced oxidative stress (ROS).
• SAB suppresses arecoline-activated tumor-promoting pathways.
• SAB enhances DNA repair and suppresses metabolic processes.

## Graphical abstract

**Figure:**
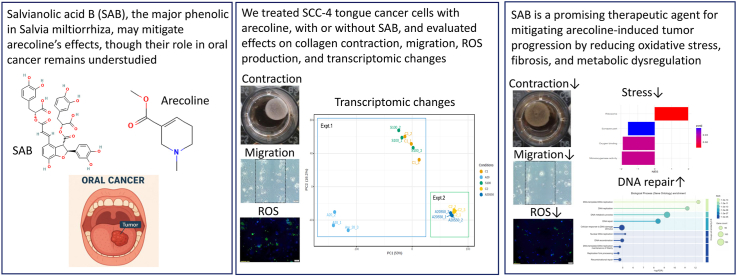

## 1 Introduction

Oral cancer remains a significant global health burden and is among the most prevalent malignancies of the head and neck (Johnson et al., 2020). Key risk factors associated with oral carcinogenesis include tobacco use, alcohol consumption, and betel quid chewing (Ko et al., 2020; Singh and Chaturvedi, 2024). Current treatment modalities, including surgery and chemoradiotherapy, often lead to severe functional impairments, affecting speech and swallowing (Johnson et al., 2020). Emerging treatment approaches, such as targeted therapies, innovative drug formulations, and natural product-based compounds, have shown promising potential for improving patient outcomes (Ko et al., 2020). In particular, natural bioactive compounds with antioxidant and anti-inflammatory properties are attracting increasing attention because of their ability to mitigate tumor progression and enhance treatment efficacy (Ko et al., 2020).

Arecoline is the principal alkaloid that is present in areca nuts, which are chewed by 10–40 % of the population worldwide, especially in South/Southeast Asia, the tropical Pacific, and East Africa (Chen et al., 2020; Ku et al., 2021; Singh and Chaturvedi, 2024). Although arecoline has medical benefits in the treatment of infection, it is cytotoxic and genotoxic and can induce oral submucous fibrosis (OSF), oral leukoplakia, and subsequent oral cancer (Ko et al., 2023; Oliveira et al., 2021; Senevirathna et al., 2023; Sharma et al., 2024; Xie et al., 2022). Accumulating evidence has revealed that arecoline stimulates inflammation, increases reactive oxygen species (ROS) levels, and activates multiple signaling pathways, including hypoxia inducible factor-1 (HIF-1), transforming growth factor beta (TGF-β), and epidermal growth factor receptor (EGFR), thereby promoting fibrosis as well as cancer initiation, proliferation, and invasion (Gocol et al., 2023; Sharma et al., 2024; Shen et al., 2020; Singh and Chaturvedi, 2024; Xie et al., 2022; Zhang et al., 2021).

Salvianolic acid B (SAB) is the most abundant water-soluble phenolic compound found in the roots and rhizomes of *Salvia miltiorrhiza* Bunge (Danshen), which is a traditional medicinal herb that is widely used in Asia for the treatment of cardiovascular diseases (Shahzadi et al., 2020; Wei et al., 2025; Xu et al., 2023). The molecular formula of SAB (C_36_H_30_O_16_) includes seven phenolic hydroxyl groups, which contribute to its potent antioxidant properties (Chen et al., 2014; Shahzadi et al., 2020; Wei et al., 2025; X. Xu et al., 2023). SAB has diverse pharmacological activities, including antioxidant (Lin et al., 2021; Zhao et al., 2011), anti-inflammatory (Shahzadi et al., 2020), anticancer (Huang et al., 2024; Liang et al., 2024; Wang et al., 2021), cardioprotective (Lin et al., 2016; Wang et al., 2011), neuroprotective (Tian et al., 2008; Wang et al., 2023), and hepatoprotective (Lin et al., 2021; F. Liu et al., 2023) effects. SAB has also been reported to attenuate oral submucous fibrosis and reverse drug resistance in colorectal and gastric cancer cells (J. Huang et al., 2024; Liang et al., 2024; Wang et al., 2021). SAB appears to have the potential to mitigate the effects of arecoline. However, the interaction between SAB and arecoline in oral cancer has been less frequently discussed in the literature. In this study, we therefore investigated the effects of arecoline, SAB, and their combination on oral cancer cells.

## 2 Materials and methods

### 2.1 Arecoline and SAB

Arecoline (98+% arecoline hydrobromide) was purchased from Acros Organics (Geel, Belgium), and SAB (95+%) was purchased from Cayman (Ann Arbor, MI, USA). With respect to reference dosages, arecoline is typically used at 10–50 μg/mL in *in vitro* studies with oral cells, with 20 μg/mL often employed to model the pathogenic mechanisms of OSF and oral squamous cell carcinoma (OSCC) (Liu et al., 2016). SAB is typically employed in *in vitro* studies on its anti-tumor effects at concentrations of 1–200 μg/mL, with 10–100 μg/mL representing the most frequently used reference range across various cancer cell lines (He et al., 2023). Accordingly, in the subsequent experiments, the negative control consisted of 0 μg/mL arecoline with 0 μg/mL SAB (untreated), the positive control consisted of 20 μg/mL arecoline with 0 μg/mL SAB, and all the other conditions were designated as treatment groups.

### 2.2 SCC-4 cell culture

The SCC-4 tongue cancer cell line was purchased from the Bioresource Collection and Research Center (BCRC) in Taiwan. These cells were harvested in Dulbecco's modified Eagle's medium/Ham's F12 (Gibco) supplemented with 10 % fetal bovine serum (Gibco), 1 % penicillin‒streptomycin‒amphotericin B (Gibco), and 400 ng/ml hydrocortisone (TargetMol), and maintained at 37 °C in a humidified 5 % CO_2_ incubator.

### 2.3 Cell viability analysis using the CCK-8 assay

The viability of SCC-4 cells that were treated with different concentrations of arecoline or SAB was evaluated using a CCK-8 assay kit (Ab228554; Abcam). In brief, SCC-4 cells were plated in 96-well plates (1 × 10^4^ cells/well) and cultured for 2 days. Afterward, the CCK-8 reagent was added to the wells according to the manufacturer's instructions, and the cells were incubated for more than 30 min. The half maximal inhibitory concentration (IC_50_) values of arecoline or SAB were calculated by measuring the absorbance at 460 nm for each sample.

### 2.4 Collagen gel contraction assay

SCC-4 cells were suspended in a solution with collagen type 1 (1 mg/ml) (3440-100-01; R&D) according to the standard protocol (Lee et al., 2018; Ngo et al., 2006). The cell-collagen mixtures (1 × 10^5^ cells/500 μl per well) were first loaded in a 24-well plate, and then, 500 μl of culture medium supplemented with arecoline (20 μg/ml) and different concentrations of SAB (10, 50 and 100 μg/ml) was added to each well 20 min after collagen polymerization. Changes in the collagen gel surface area were captured by imaging every day and quantified by ImageJ (version 1.53k) to determine the degree of collagen contraction.

### 2.5 Wound healing assay

SCC-4 cells (2 × 10^5^ cells/2 ml per well) were seeded in 6-well culture plates with different concentrations of arecoline/SAB and incubated for 1 day. Afterward, a straight-line wound was made on the middle portion of each well with a 200-μl plastic pipette tip. After incubation for one additional day, cell migration toward the wounded area was recorded with a microscope and quantified by ImageJ (version 1.53k).

### 2.6 Intracellular ROS assay

Intracellular ROS levels in SCC-4 cells were determined using the fluorescent probe 2,7-dichlorofluorescein diacetate (DCFH-DA) as previously described (LeBel et al., 1992). SCC-4 cells (1 × 10^4^ cells/well) were seeded in a 96-well plate and cultured with different concentrations of arecoline/SAB for 1 day. Afterward, these cells were incubated with the intracellular ROS fluorescent probe (DCFH-DA; 10 μM) for 30 min and the nuclear fluorescent dye 4′,6-diamidino-2-phenylindole (DAPI; 1 ng/ml) for 5 min according to the standard protocol (Chen et al., 2022). The fluorescence intensities (ROS levels) were observed with an inverted fluorescence microscope (Olympus, Japan) and quantified using Olympus cellSens software (version 1.17).

### 2.7 RNA sequencing (RNA-seq) and data processing

SCC-4 cells (2 × 10^6^ cells/well) were seeded in 10-cm petri dishes and cultured with different concentrations of arecoline/SAB for 2 days. Total RNA was extracted from the cells with TRIzol (Invitrogen). The RNA concentrations and integrity were measured using a NanoDrop One spectrophotometer (Thermo Fisher) and a 2100 Bioanalyzer instrument (Agilent), respectively. An Illumina Stranded mRNA Prep Ligation Kit was used to construct the total RNA libraries. Afterward, RNA-seq with 150 paired-end base pairs was executed using the Illumina NovaSeq 6000 platform.

The raw RNA-seq data were processed by quality control (FastQC), adaptor trimming (BBDuk), and gene expression quantification (Salmon) (Patro et al., 2017). The matrix of gene expression (counts) was further processed utilizing the ExpressAnalyst platform (https://www.expressanalyst.ca/ExpressAnalyst/home.xhtml) (Liu et al., 2023). The raw count data were normalized by log2 counts per million transformations, and the differentially expressed genes (DEGs) were identified using the DESeq2 algorithm. Gene set enrichment analysis (GSEA) was performed using terms from the Kyoto Encyclopedia of Genes and Genomes (KEGG), Gene Ontology (GO), and sequence binding motifs, which are among the most widely used and well-established resources for pathway and functional annotation. KEGG provides comprehensive and well-curated signaling and metabolic pathways, GO offers a hierarchical framework for classifying genes according to biological processes, molecular functions, and cellular components, and sequence binding motifs allow the identification of potential transcriptional regulatory mechanisms. Over-representation analysis (ORA) and protein‒protein interaction (PPI) network functional enrichment analysis were performed utilizing the g:Profiler (https://biit.cs.ut.ee/gprofiler/gost) and STRING (https://string-db.org/) platforms, respectively (Kolberg et al., 2023; Szklarczyk et al., 2019). g:Profiler is currently among the most widely used tools for ORA, with the advantage of identifying potential driver pathways while reducing redundancy from similar GO terms. The normalized gene expression in terms of transcripts per million (TPM) was obtained directly by the Salmon algorithm, and corresponding heatmaps were generated utilizing Morpheus (https://software.broadinstitute.org/morpheus/).

### 2.8 Statistical analysis

Statistical analyses were performed using GraphPad Prism (version 9), ExpressAnalyst, g:Profiler, or STRING. The IC_50_ values were estimated via nonlinear regression, and the corresponding cytotoxicity plots were constructed using GraphPad Prism. The levels of collagen contraction, cell migration, and intracellular ROS were compared using independent T tests (2 groups) or analysis of variance (ANOVA) with post hoc tests (3 or more groups). With respect to RNA-seq, principal component analysis (PCA) plots, GSEA horizontal bar charts, ORA plots, Venn diagrams, and PPI enrichment plots were generated via ExpressAnalyst, the ggplot2 package in R (version 4.4.2), g:Profiler, an online tool (https://bioinformatics.psb.ugent.be/webtools/Venn/), and STRING, respectively. An adjusted p value was used in the RNA-seq analysis. A statistically significant result was considered when the corresponding p value or adjusted p value was less than 0.05.

## 3 Results

### 3.1 Viability of SCC-4 tongue cancer cells treated with arecoline or SAB

**Fig. 1:**
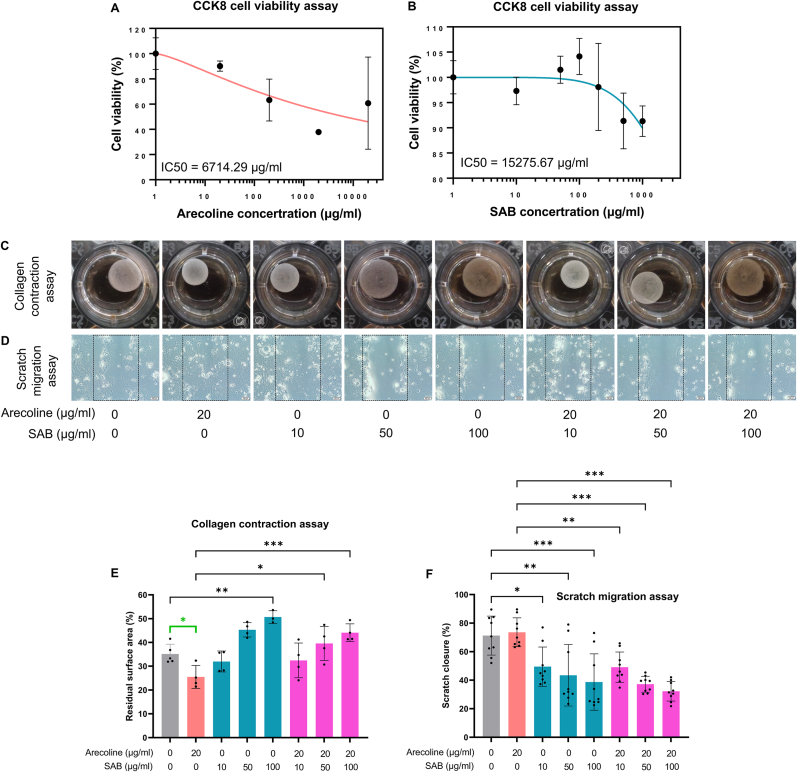
Effects of arecoline and SAB on cell viability, collagen contraction, and migration in SCC-4 oral cancer cells. A, B. Effects on the viability of SCC-4 cells treated with arecoline (A) or SAB (B), as measured by a CCK-8 assay (n = 3 per concentration); C, E. Collagen contraction of SCC-4 cells treated with arecoline or SAB after 1 week of incubation, as measured by a collagen contraction assay (n = 3 per group) (C shows representative images, and E shows a statistical bar chart); D, F. Migration of SCC-4 cells treated with arecoline or SAB after 1 day of incubation, as measured by a wound healing assay (n = 3 per group) (D shows representative images, and F shows a statistical bar chart). The negative control was 0 μg/ml arecoline +0 μg/ml SAB, the positive control was 20 μg/ml arecoline +0 μg/ml SAB, and all the other conditions were treatment groups. SAB: salvianolic acid B; CCK8: cell counting kit 8; IC50: half maximal inhibitory concentration; Comparison lines: green, two-sample *t*-test; black, analysis of variance (ANOVA); ∗P < 0.05; ∗∗P < 0.01; ∗∗∗P < 0.001.

SCC-4 cells were treated with varying concentrations of arecoline or SAB for 2 days. The results of the CCK8 assay revealed IC50 values of 6714.29 μg/ml for arecoline and 15275.67 μg/ml for SAB (Fig. 1A and B). Accordingly, on the basis of the reference dose ranges reported in the literature and described in the Methods, arecoline was applied at 20 μg/mL and SAB at 10, 50, and 100 μg/mL in the following experiments, which resulted in minimal cytotoxicity to SCC-4 cells. The negative control consisted of untreated cells (0 μg/mL arecoline and 0 μg/mL SAB), the positive control comprised cells treated with 20 μg/mL arecoline alone, and all the other conditions were defined as treatment groups.

### 3.2 SAB mitigated the effect of arecoline on promoting collagen contraction in SCC-4 cells

The collagen gel contraction assay demonstrated a progressive reduction in surface area over one week of incubation, with the SCC-4 cell–collagen mixture contracting from 100 % to 35.14 ± 4.09 % (Fig. 1C). Treatment with arecoline (20 μg/ml) reduced the final residual surface area to 25.48 ± 4.84 %, which was statistically significant in the two-sample t tests (P = 0.01), despite not reaching significance in the ANOVA post hoc analyses (Fig. 1E). Conversely, SAB (100 μg/ml) treatment significantly increased the residual surface area to 50.68 ± 2.67 %, indicating its potential to attenuate collagen contraction. Moreover, in the presence of arecoline (20 μg/ml), SAB treatment (50 and 100 μg/ml) restored the residual surface area to 39.54 ± 7.12 and 44.08 ± 3.74 %, respectively. Compared with arecoline treatment alone, cotreatment with arecoline and SAB resulted in a significant difference according to ANOVA post hoc tests. These results suggest that SAB effectively counteracts the pro-fibrotic effects of arecoline, mitigating collagen contraction in SCC-4 cells. These findings highlight the potential therapeutic role of SAB in reducing fibrosis-associated tumor microenvironment alterations, which may be relevant for oral cancer treatment strategies.

### 3.3 SAB decreased SCC-4 cell migration regardless of the presence of arecoline

As shown in Fig. 1D and F, treatment with arecoline (20 μg/ml) alone did not significantly affect the wound healing rate, suggesting that arecoline does not directly increase SCC-4 cell migration. In contrast, treatment with SAB alone resulted in a dose-dependent decrease in wound healing, with significant inhibition observed at 50 and 100 μg/ml (P < 0.01). Notably, even in the presence of arecoline (20 μg/ml), SAB maintained its antimigratory effects, significantly reducing wound healing at concentrations of both 50 and 100 μg/ml (P < 0.01). These findings indicate that SAB effectively suppresses SCC-4 cell migration independent of arecoline exposure, suggesting its potential role in inhibiting oral cancer cell motility and metastasis.

### 3.4 SAB relieved the oxidative stress generated by arecoline in SCC-4 cells

**Fig. 2:**
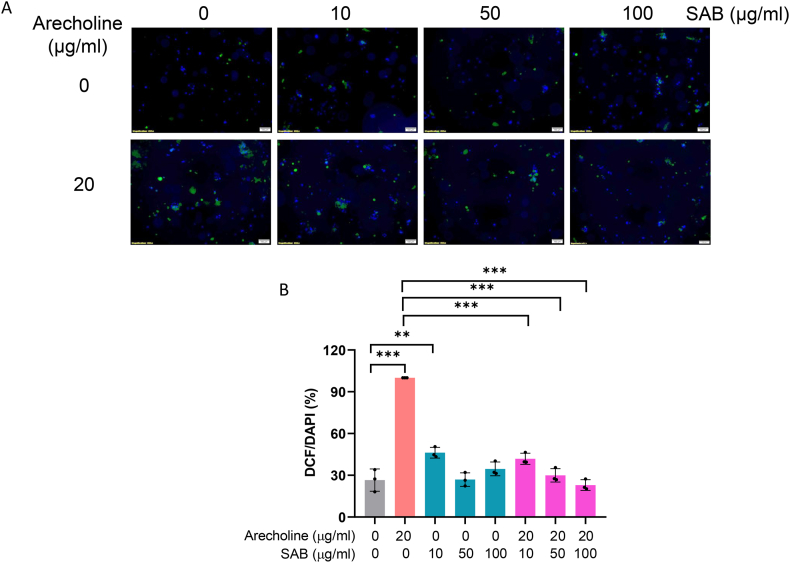
Intracellular ROS levels in SCC-4 oral cancer cells treated with arecoline or SAB after 1 day of incubation, assessed using a fluorescent DCFH-DA probe. A. Representative images; B. Statistical bar chart (n = 3 per group). The negative control was 0 μg/ml arecoline +0 μg/ml SAB, the positive control was 20 μg/ml arecoline +0 μg/ml SAB, and all the other conditions were treatment groups. ROS: reactive oxygen species; SAB: salvianolic acid B; DCFH-DA: 2,7-dichlorofluorescein diacetate; DAPI: 4′,6-diamidino-2-phenylindole; Comparison lines: analysis of variance (ANOVA); ∗P < 0.05; ∗∗P < 0.01; ∗∗∗P < 0.001.

An intracellular ROS assay demonstrated that arecoline (20 μg/ml) significantly increased ROS levels in SCC-4 cells (Fig. 2A and B). Interestingly, treatment with SAB (10 μg/ml) alone also induced a mild increase in ROS production. However, when SAB was coadministered with arecoline, a significant reduction in ROS levels was observed compared with those in the group treated with arecoline alone (P < 0.01), suggesting that SAB has a protective effect against arecoline-induced oxidative stress. Notably, the ROS-reducing effect of SAB was dose dependent, with 50 and 100 μg/ml SAB resulting in the most significant suppression of arecoline-induced ROS accumulation in SCC-4 cells (P < 0.001). These findings suggest that SAB effectively mitigates oxidative stress in SCC-4 cells, potentially counteracting the protumorigenic effects of ROS in oral cancer.

### 3.5 SAB alleviated the tumor-promoting pathways activated by arecoline in SCC-4 cells

**Fig. 3:**
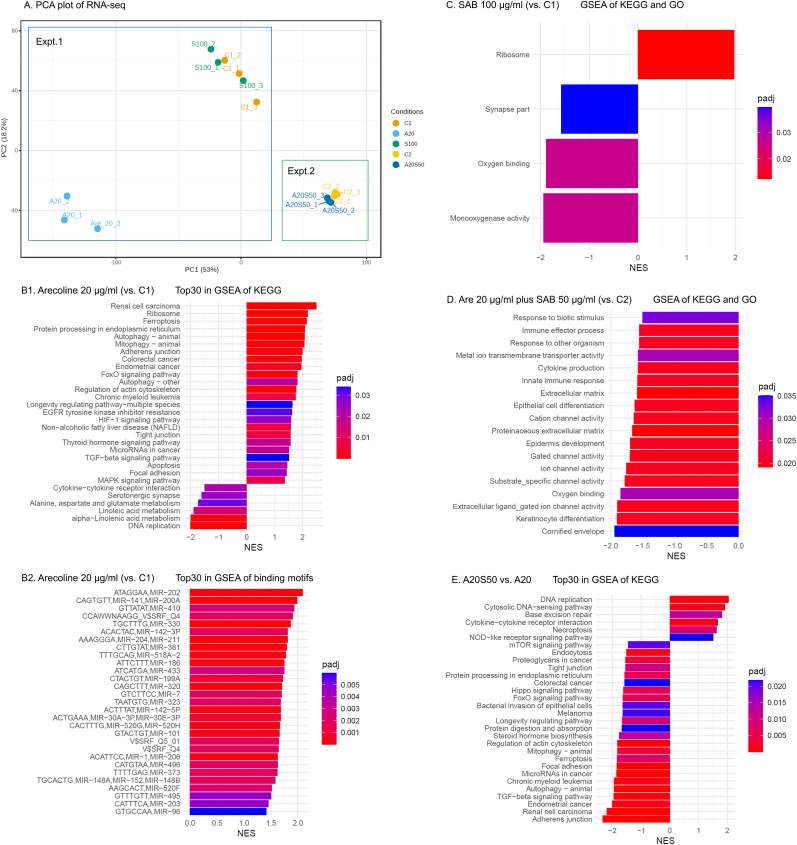
RNA-seq-based PCA and GSEA analyses of SCC-4 cells following 2 days of treatment with arecoline or SAB. A. PCA plot; B1**–**2. Top 30 GSEA-enriched KEGG pathways (B1) and transcription factor binding motifs (B2) identified in SCC-4 cells treated with arecoline (20 μg/ml) compared with those in C1 (untreated negative control) (n = 3 per group, Expt. 1); C. Significant GSEA-enriched KEGG and GO pathways identified in SCC-4 cells treated with SAB (100 μg/ml) compared with those in C1 (untreated negative control) (n = 3 per group, Expt. 1); D. Significant GSEA-enriched KEGG and GO pathways identified in SCC-4 cells treated with arecoline (20 μg/ml) plus SAB (50 μg/ml) compared with those in C2 (untreated negative control) (n = 3 per group, Expt. 2). E. Top 30 GSEA-enriched KEGG pathways identified in SCC-4 cells treated with arecoline (20 μg/ml) plus SAB (50 μg/ml) (Expt. 2) relative to cells treated with arecoline (20 μg/ml) alone (Expt. 1) (n = 3 per group). RNA-seq: RNA sequencing; PCA: principal component analysis; GSEA: gene set enrichment analysis; SAB: salvianolic acid B; KEGG: Kyoto Encyclopedia of Genes and Genomes; GO: Gene Ontology; Expt.: experiment; NES: normalized enrichment score; padj: adjusted p value; C1: control 1; C2: control 2; A20: 20 μg/ml arecoline; S100: 100 μg/ml SAB; A20S50: 20 μg/ml arecoline plus 50 μg/ml SAB.

RNA-seq analysis was conducted to examine the molecular effects of arecoline (20 μg/ml) on SCC-4 cells. PCA plots demonstrated distinct clustering between arecoline-treated and untreated SCC-4 cells, indicating significant transcriptomic alterations (Fig. 3A). GSEA further revealed that arecoline significantly upregulated key profibrotic and tumor-associated pathways, including those related to adherens junctions, tight junctions, focal adhesion, TGF-β signaling, EGFR tyrosine kinase inhibitor resistance, HIF-1 signaling, MAPK signaling, and microRNAs in cancer, all of which contribute to tumor progression and fibrosis (Fig. 3B). Additionally, arecoline enhanced ferroptosis-related pathways, suggesting an increase in intracellular lipid peroxidation, which may further exacerbate oxidative stress and carcinogenesis.

In contrast, treatment with SAB (100 μg/ml) or SAB (50 μg/ml) in combination with arecoline (20 μg/ml) resulted in gene expression profiles similar to those of untreated SCC-4 cells, as shown by PCA (Fig. 3A). SAB significantly downregulated pathways related to oxygen binding and monooxygenase activity, indicating its potential role in reducing oxidative stress in SCC-4 cells (Fig. 3C). Notably, in the presence of SAB, arecoline-induced activation of profibrotic and tumor-promoting pathways was markedly suppressed, and pathways associated with oxygen-binding activity were further downregulated (Fig. 3D). Moreover, compared with arecoline treatment alone, cotreatment with SAB significantly decreased the expression of genes involved in adherens junctions, tight junctions, focal adhesion, TGF-β signaling, and microRNAs in cancer, supporting its antifibrotic and antitumorigenic effects (Fig. 3E).

Our findings suggest that SAB effectively mitigates arecoline-induced oxidative stress and suppresses key pathways associated with fibrosis and tumor progression in SCC-4 cells, highlighting its potential therapeutic role in oral cancer prevention and treatment.

### 3.6 Antistress activities of SAB abrogated the influence of arecoline in SCC-4 cells

**Fig. 4:**
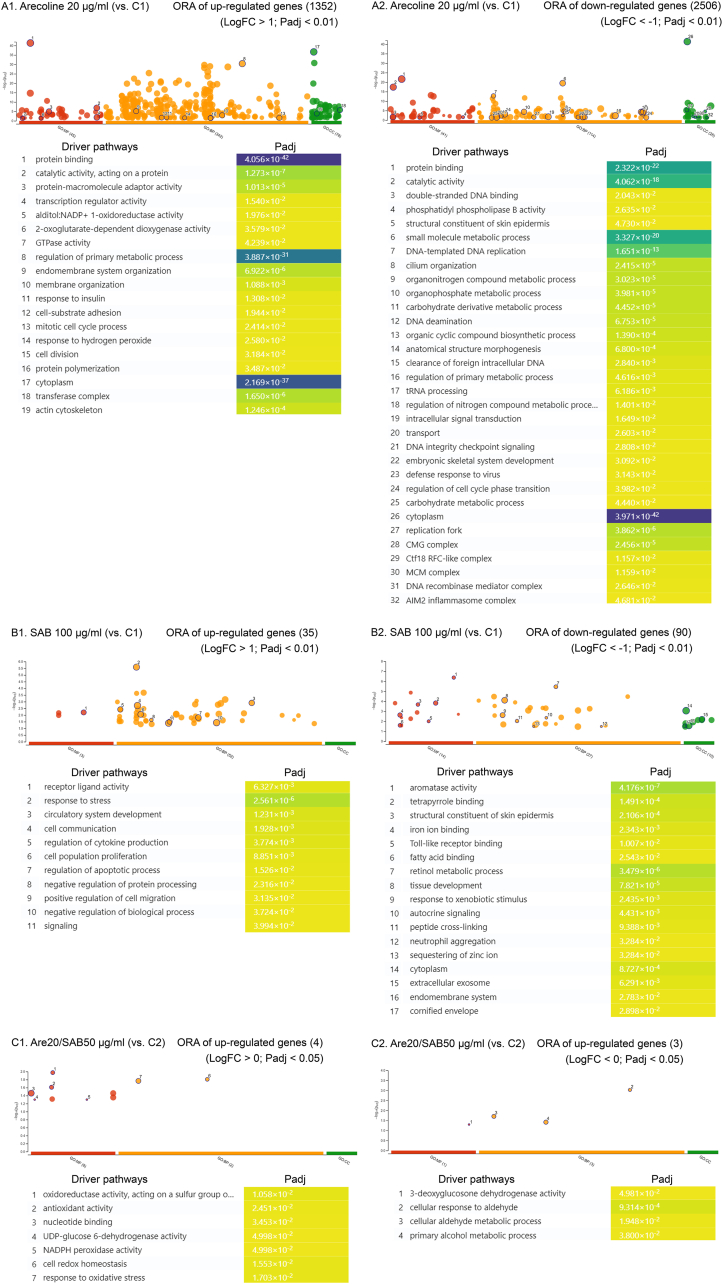
RNA-seq–derived ORA of SCC-4 cells following 2 days of treatment with arecoline or SAB. A1**–**2. Significant enrichment of GO driver pathways among upregulated genes (A1) and downregulated genes (A2) in SCC-4 cells treated with arecoline (20 μg/ml) compared with C1 (untreated negative control) (n = 3 per group, Expt. 1); B1–2. Significant enrichment of GO driver pathways among upregulated genes (B1) and downregulated genes (B2) in SCC-4 cells treated with SAB (100 μg/ml) compared with C1 (untreated negative control) (n = 3 per group, Expt. 1); C1**–**2. Significant enrichment of GO driver pathways among upregulated genes (C1) and downregulated genes (C2) in SCC-4 cells treated with arecoline (20 μg/ml) plus SAB (50 μg/ml) compared with C2 (untreated negative control) (n = 3 per group, Expt. 2). RNA-seq: RNA sequencing; ORA: over-representation analysis; SAB: salvianolic acid B; GO: Gene Ontology; MF: molecular function; BP: biological process; CC: cellular component; Expt.: experiment; FC: fold change; Padj: adjusted P value; C1: control 1; C2: control 2; Are20: 20 μg/ml arecoline.

In addition to PCA and GSEA, RNA-seq-based ORA was performed on both upregulated and downregulated genes. Arecoline treatment significantly upregulated pathways associated with transcription regulator activity, GTPase activity, the mitotic cell cycle, the response to hydrogen peroxide, and cell-substrate adhesion, all of which contribute to tumor growth and progression (Fig. 4A). Additionally, arecoline exerted a dual regulatory effect on metabolic processes, influencing both upregulated and downregulated metabolic pathways (Fig. 4A), suggesting its broad impact on cellular metabolism and tumorigenesis.

On the other hand, SAB treatment significantly increased the expression of genes associated with cellular stress responses and apoptosis but decreased the expression of genes related to autocrine signaling and extracellular exosome production, which might hinder cancer growth (Fig. 4B). Notably, in the presence of arecoline, SAB could still reinforce the pathways involved in antioxidant activity and the response to oxidative stress (Fig. 4C), highlighting its potential role in counteracting arecoline-induced oxidative damage. These findings support the hypothesis that SAB exerts antistress effects and abrogates the tumor-promoting effect of arecoline in SCC-4 cells, suggesting its potential for use in oral cancer prevention and treatment.

### 3.7 SAB enhanced DNA repair and reduced metabolic processes in the presence of arecoline

**Fig. 5:**
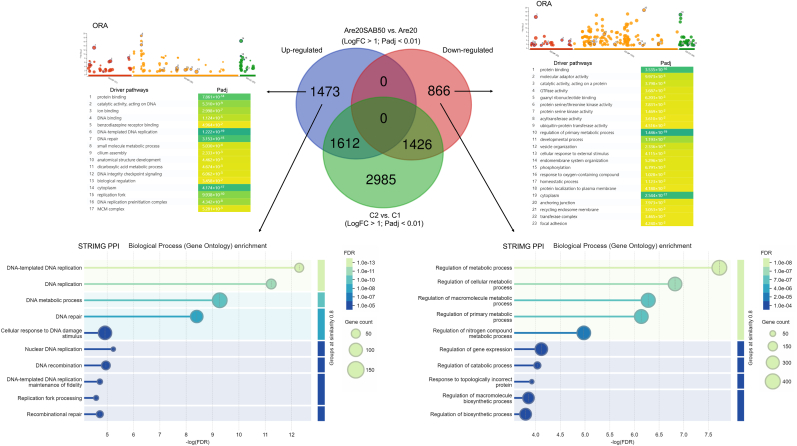
RNA-seq–based ORA and STRING PPI analyses of SCC-4 cells following 2 days of treatment with SAB in the presence of arecoline. Comparisons were made between cells treated with arecoline (20 μg/ml) plus SAB (50 μg/ml) (Expt. 2) and cells treated with arecoline (20 μg/ml) alone (Expt. 1), after adjusting to minimize background differences between C2 (Expt. 2) and C1 (Expt. 1). RNA-seq: RNA sequencing; ORA: over-representation analysis; PPI: protein–protein interaction; Expt.: experiment; C1: control 1; C2: control 2; Are20: 20 μg/ml arecoline; Are20SAB50: 20 μg/ml arecoline plus 50 μg/ml SAB; FC: fold change; Padj: adjusted P value; FDR: false discovery rate.

To minimize background variations, the RNA-seq-derived DEGs that were identified between control 1 and control 2 SCC-4 cells were excluded from the DEG analysis between the arecoline/SAB (Are20/SAB50, background: control 2) group and the arecoline-alone (Are20, background: control 1) group (Fig. 5). This approach ensured that the observed changes in gene expression were specifically attributed to SAB treatment rather than intrinsic differences in background.

Both ORA and STRING PPI analysis revealed that in the presence of arecoline, SAB enhanced DNA repair pathways while suppressing metabolic processes, representing a shift that may be beneficial for impeding cancer progression. The upregulation of DNA repair pathways suggests that SAB may counteract arecoline-induced genetic damage, thereby reducing the potential for mutagenesis and tumorigenesis. Moreover, the downregulation of metabolic pathways is consistent with a tumor-suppressive effect, as metabolic reprogramming is a hallmark of cancer progression (Fig. 5). Our findings suggest that SAB not only mitigates the genomic instability induced by arecoline but also disrupts metabolic pathways that are essential for tumor growth, reinforcing its potential therapeutic role in oral cancer treatment.

### 3.8 Inverse effects of arecoline and SAB on the expression of multiple genes in pathways related to fibrosis and proliferation

**Fig. 6:**
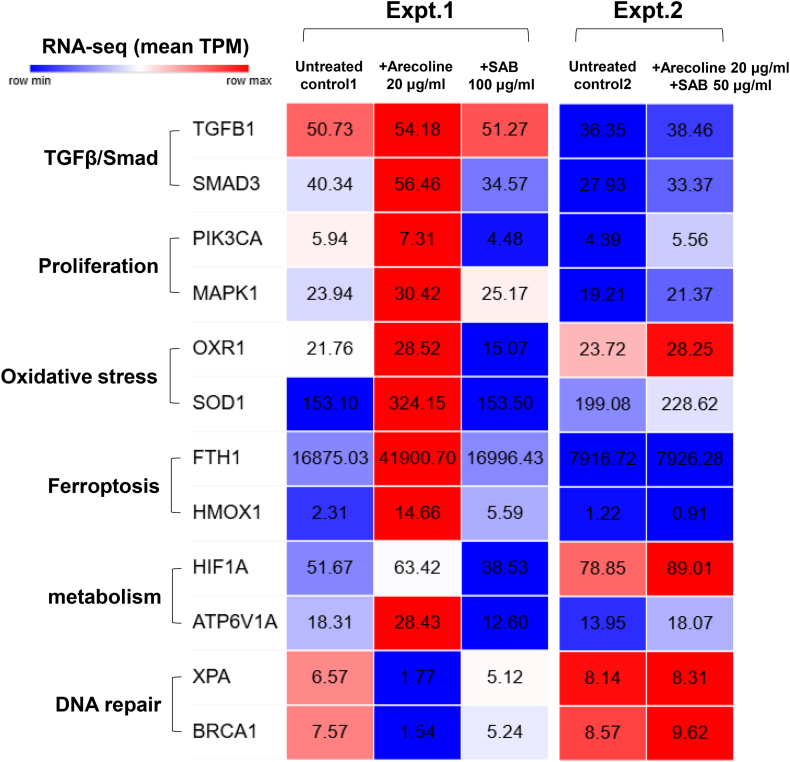
RNA-seq–based tumor proliferation–associated gene expression (unit: mean TPM) in SCC-4 cells following 2 days of treatment with arecoline or SAB (simplified version). RNA-seq: RNA sequencing; TPM: transcript per million; SAB: salvianolic acid B; Expt.: experiment.

RNA-seq quantification using units of TPM revealed that arecoline significantly upregulated the expression of genes involved in the TGFβ/Smad signaling pathway (*TGFB1/2*, *TGFBR1/3*, and *SMAD3*). Additionally, arecoline increased the expression of genes related to cell proliferation (*PIK3CA*, *MAPK1*, *RAF1*, and *SOS1*), oxidative stress (*MAOA*, *OXR1*, *SOD1*, and *GSR*), ferroptosis (*FTH1*, *SLC40A1*, *NFE2L2*, and *HMOX1*), and metabolism (*HIF1A* and *ATP6V1A)* but decreased the expression of genes involved in DNA repair (OGGA, XPA, and BRCA1/2) (experiment 1) (Fig. 6 and Supplementary Fig. S1). In contrast, SAB treatment reversed these effects, downregulating the expression of genes involved in the TGFβ/Smad signaling pathways (*TGFB2*, *TGFBR1/3*, and *SMAD3*). SAB also suppressed the expression of proliferation-related genes *(PIK3CA* and *SOS1*), genes involved in oxidative stress (*MAOA* and *OXR1*), and genes involved in metabolism (*HIF1A* and *ATP6V1A)* (experiment 1). Furthermore, when combined with SAB, the effects of arecoline on those pathways became insignificant, which supported the protective effects of SAB on cells against environmental stress (experiment 2).

These findings demonstrate that SAB counteracts the profibrotic and pro-proliferative effects of arecoline, highlighting its potential therapeutic value in preventing fibrosis-driven tumor progression in oral cancer.

## 4 Discussion

This study demonstrated that arecoline promotes collagen contraction, induces ROS accumulation, and activates multiple tumor-promoting pathways, including adhesion, fibrosis, proliferation, and ferroptosis, in SCC-4 oral cancer cells. In contrast, SAB effectively counteracts these effects, significantly reducing collagen contraction, inhibiting cell migration, and mitigating oxidative stress. Notably, even in the presence of arecoline, SAB treatment not only suppressed fibrosis-related processes but also modulated metabolic activity and enhanced pathways associated with antioxidant responses and DNA repair. These findings suggest that SAB may be a therapeutic agent that counteracts the profibrotic and tumor-promoting effects of arecoline in oral cancer.

Previous studies have shown that arecoline activates multiple oncogenic pathways, including those involving TGF-β, HIF-1, MAPK, EGFR, and oxidative stress, thereby facilitating fibrosis, epithelial–mesenchymal transition (EMT), and cancer progression (Gocol et al., 2023; G. Huang et al., 2024; Senevirathna et al., 2023; Singh and Chaturvedi, 2024). Arecoline has been shown to induce ROS production in both normal oral epithelial cells and OSCC cells (Shih et al., 2021). Arecoline was also found to increase collagen production in oral keratinocytes and fibroblasts, and collagen contraction is a key feature of cancer development, influencing tumor growth and fibrosis, as well as tumor invasion, migration, and metastasis (Lee et al., 2018; Uzawa et al., 2024; Xia et al., 2009). Our findings confirm these results and further reveal that arecoline induces ferroptosis, which is a process rarely discussed in the context of oral cancer. Ferroptosis is a nonapoptotic mechanism of programmed cell death that is characterized by iron-dependent lipid peroxidation, which requires the accumulation of lipid ROS (Cao and Dixon, 2016; Dixon and Olzmann, 2024; Stockwell et al., 2017; Wang et al., 2024; Zhou et al., 2024). Although ferroptosis may suppress tumor proliferation, the oxidative stress accompanying this process can induce further damage to both normal and cancer cells, potentially exacerbating carcinogenesis (Kuang et al., 2020; Wang et al., 2024; Zhou et al., 2024).

Antioxidants have been explored as agents that can mitigate arecoline-induced carcinogenesis, with N-acetylcysteine and glutathione previously reported to reduce oxidative damage (Ko et al., 2023). The natural antioxidants quercetin, curcumin, resveratrol, and naringenin have been reported to have promising inhibitory effects on oral cancer and have been suggested to be suitable for modulating the tumor microenvironment (Budi and Farhood, 2023; Prakash et al., 2021). Our study extends this understanding by demonstrating that SAB effectively alleviates arecoline-induced oxidative stress, modulates the tumor microenvironment, and suppresses fibrosis-related pathways, further supporting its potential for use in treating oral cancer. Compared with Danshensu, vitamin C, and *Ginkgo biloba* extract (EGb 761), SAB has been shown to have stronger antioxidant activity in various *in vitro* assays (Liu et al., 2006; Zhao et al., 2008). These findings highlight the promising potential of SAB as a natural antioxidant for therapeutic applications, particularly in oxidative damage-related conditions, such as arecoline-induced oral cancer.

SAB, which is a natural polyphenol derived from *S*. *miltiorrhiza*, is a potent antioxidant owing to its polyphenolic structure, which underlies its wide application in the treatment of myocardial infarction, atherosclerosis, stroke, diabetes, cancer, and fibrosis-related diseases affecting the heart, kidney, liver, lung, skin, and oral cavity (He et al., 2023; Liang et al., 2024). Its strong antioxidant activity is considered a central mechanism underlying its protective effects, while additional regulatory actions on cellular signaling pathways contribute to its proapoptotic, antiangiogenic, and antifibrotic effects across multiple cancer types. Specifically, SAB has been shown to regulate glycolysis through the PI3K/AKT/HIF-1α signaling pathway, thereby inhibiting metabolic reprogramming and tumor growth in a hamster oral cancer model (Wei et al., 2018). Studies have also demonstrated that SAB inhibits HIF-1α and VEGF expression, thereby suppressing tumor-associated neovascularization in oral cancer cell lines (Yang et al., 2011). Moreover, SAB was found to suppress COX-2 expression and induce apoptosis in head and neck cancer xenograft mouse models, supporting its potential as a COX-2-targeting chemopreventive agent (Hao et al., 2009). In mouse models of breast cancer, SAB has been shown to reduce oxidative stress, induce apoptosis, inhibit inflammation and angiogenesis, and thereby hinder tumor growth (Katary et al., 2019). In mouse models of colon cancer, SAB was found to reduce multidrug resistance and inhibit tumor invasion and growth (Guo et al., 2018). Multiple studies have shown that SAB appears to inhibit multiple targets, affecting key pathways, including the PI3K/AKT, mTOR, MAPK, and Hippo/YAP pathways, thereby reducing cellular inflammation and promoting autophagy (Jing et al., 2016; Wang et al., 2021; W. Xu et al., 2023; Yang et al., 2024). The significant inhibitory effect of SAB on the MAPK/Smad2/3 pathway has also been confirmed in lung and liver cancer models (Han et al., 2022; W. Xu et al., 2023). SAB has also been reported to inhibit TGF-β/Smad signaling to reduce fibrosis and attenuate ROS levels, thereby suppressing atherosclerosis in cardiovascular disease models (He et al., 2023; Liang et al., 2024; Shahzadi et al., 2020; X. Xu et al., 2023). These reported mechanisms are consistent with our findings, further supporting the antioxidant, anticancer, anti-inflammatory, and antifibrotic properties of SAB. Additionally, our study revealed previously underexplored roles of SAB in facilitating DNA repair and suppressing metabolic activity, contributing to its protective effects against arecoline-induced oncogenesis. These findings provide new insights into the therapeutic potential of SAB in oral cancer treatment.

Regarding safety, SAB is generally well tolerated; doses up to 300 mg/kg caused no maternal or embryonic toxicity in rats, a dose of 100 mg/kg did not adversely affect embryo–fetus development or induce genotoxicity, and toxicity was observed only at high doses (750 mg/kg), indicating a wide safety margin for potential clinical use (He et al., 2023). A phase 1 clinical study in healthy Chinese volunteers demonstrated that single doses of SAB up to 300 mg and multiple doses up to 250 mg for 5 days were well tolerated, with only minor and reversible adverse events observed, including elevated ALT (4 %), bilirubin (2 %), CK-MB (2 %), and brain natriuretic peptide (8 %), as well as increased dizziness (2 %), and chest discomfort (2 %) (Cheng et al., 2023). As for drug–drug interactions, clinical data suggested that, when combined with aspirin, SAB prolonged its half-life and further suppressed CD62p (a platelet activation marker), while overall having a neutral effect on platelet inhibition compared with aspirin alone (Cao et al., 2021). SAB may exhibit enhanced therapeutic effects when combined with other natural compounds or antioxidants, such as tanshinone, ferulic acid, or ginsenoside Rg1, as coadministration can improve its pharmacokinetics and synergistically increase tissue-protective activities (He et al., 2023; Liang et al., 2024). Future studies should investigate the potential synergistic effects of SAB in combination with existing chemotherapeutic agents or immunotherapies, as such combination treatments may enhance therapeutic efficacy while reducing toxicity and resistance.

Despite its promising therapeutic properties and favorable safety profile, the clinical application of SAB is limited by its poor bioavailability and low systemic delivery efficiency (He et al., 2023). Recent advances in phospholipid nanoparticle-based drug delivery systems (SAB–PLC–NPs) have demonstrated the enhanced cellular uptake and anticancer efficacy of SAB in head and neck cancers (Li et al., 2016). *In vivo* studies have confirmed that nanoformulated SAB significantly reduces tumor incidence and suppresses tumor growth in oral cancer models (Liu et al., 2022). Future research should focus on optimizing drug delivery strategies to improve the pharmacokinetics and therapeutic efficacy of SAB for use in oral cancer treatment. In addition, SAB also has poor chemical stability, which may limit its clinical applications (He et al., 2023). Enhancing the stability of SAB through innovative formulation strategies may be crucial for its successful clinical translation.

The primary limitation of this study is that all the experiments were performed using a 2D cell culture model, which may not accurately recapitulate the complexity of the *in vivo* tumor microenvironment. A second limitation is that only a single cancer cell line was used. Future investigations should incorporate 3D organoid models, *in vivo* animal studies, and multiple cancer cell lines to further validate these findings and explore the clinical relevance of SAB in oral cancer treatment. The third limitation is the lack of protein-level investigations using techniques such as Western blotting or enzyme-linked immunosorbent assay (ELISA) to examine molecular changes. However, we conducted several protein-level functional assays, including collagen contraction, wound healing, and intracellular ROS assays, to evaluate the functional consequences of protein activity, and we employed RNA-seq to understand global gene expression changes. Future studies should incorporate protein-level validation using Western blotting or ELISA to complement functional assays and RNA-seq, providing more precise insight into the molecular mechanisms of SAB.

In conclusion, this study provides novel insights into the oncogenic role of arecoline in oral cancer, demonstrating its ability to induce collagen contraction, increase ROS generation, and activate tumor-promoting pathways related to fibrosis, proliferation, and ferroptosis. Importantly, we identified SAB as a potential therapeutic agent that can counteract these effects. SAB effectively suppressed collagen contraction, inhibited SCC-4 cell migration, alleviated oxidative stress, and downregulated the activity of profibrotic and tumor-associated pathways, including the TGF-β/Smad, EGFR, and MAPK signaling pathways. Additionally, SAB enhanced antioxidant activity, DNA repair mechanisms, and metabolic regulation, further supporting its antifibrotic and antitumorigenic properties. These findings highlight the therapeutic potential of SAB in mitigating arecoline-induced oral carcinogenesis through the targeting of fibrosis, oxidative stress, and metabolic dysregulation. On the basis of its broad pharmacological activities and our findings, SAB shows potential for use as a therapeutic agent against arecoline-induced oral cancer progression and holds promise for the future development of drugs for use in oral cancer therapy. Further *in vivo* studies and clinical trials are warranted to validate its efficacy, optimize its stability and bioavailability, and explore its potential integration into existing treatment strategies.

## Informed consent

Not applicable.

## Author contributions

Conceived and designed the research: Yi-Ling Ye, Chi-Maw Lin and Li-Shian Shi. Collected the data: Hsuan-Yin Tung and Chi-Maw Lin. Analyzed the data: Hsuan-Yin Tung and Chi-Maw Lin. Wrote and edited the paper: Li-Shian Shi and Chi-Maw Lin. Complete correspondence: Chi-Maw Lin and Li-Shian Shi.

## D ata availability

The data generated in this study are available within the article and its supplementary data files. Further information is available from the corresponding author upon request.

## Ethical approval and ethical standards

Research using established human cell lines is exempt from ethical review.

## Credit author statment

**Hsuan-Yin Tung**: Investigation, Formal analysis, Resources, Writing - Original Draft, Writing - Review & Editing, Visualization **Yi-Ling Ye**: Project administration, Funding acquisition, Conceptualization, Supervision, Validation **Chi-Maw Lin**: Conceptualization, Methodology, Software, Formal analysis, Investigation, Resources, Writing - Original Draft, Writing - Review & Editing, Visualization **Li-Shian Shi**: Data Curation, Writing - Original Draft, Writing - Review & Editing, Funding acquisition, Project administration, Supervision, Validation.

## Funding

This study was financially supported by a project from 10.13039/501100005762National Taiwan University Hospital, Yun-Lin Branch (grant number 10.13039/501100022294NTUHYL 112-C012).

## Declaration of competing interest

The authors declare that they have no known competing financial interests or personal relationships that could have appeared to influence the work reported in this paper.

## Data Availability

Data will be made available on request.

## Appendix A Supplementary data

Supplementary Figure S1. RNA-seq-based tumor proliferation-associated gene expression (unit: TPM) in SCC-4 cells following 2 days of treatment with arecoline or SAB (full version). RNA-seq: RNA sequencing; TPM: transcript per million; SAB: salvianolic acid B; Expt.: experiment.Multimeida component 1
