# Supplementary material for: Salvianolic acid B decreases oxidative stress and alleviates the tumor-promoting effects of arecoline in oral cancer
Source: Curr Res Pharmacol Drug Discov. 2025 Nov 29;9:100241. doi: 10.1016/j.crphar.2025.100241 (PMC12719042; doi:10.1016/j.crphar.2025.100241)
Supplement: Supplementary file 1 — Supplementary Figure S1. RNA-seq-based tumor proliferation-associated gene expression (unit: TPM) in SCC-4 cells following 2 days of treatment with arecoline or SAB (full version). RNA-seq: RNA sequencing; TPM: transcript per million; SAB: salvianolic acid B; Expt.: experiment.Multimeida component 1 [file mmc1.pdf]

RNA-seq (unit:TPM)

row min — row max

		Expt.1									Expt.2					
		Untreated control1			+Arecoline 20 μg/ml			+SAB 100 μg/ml			Untreated control2			+Arecoline 20 μg/ml +SAB 50 μg/ml		
	id	C1_1	C1_2	C1_3	A20_1	A20_2	A20_3	S100_1	S100_2	S100_3	C2_1	C2_2	C2_3	A20S50_1	A20S50_2	A20S50_3
TGFβ/Smad	TGFB1	52.82	48.75	50.61	48.52	57.69	56.32	50.53	53.33	49.95	33.50	39.63	35.93	36.91	38.67	39.80
	TGFB2	14.74	11.08	15.01	42.52	49.36	20.63	8.91	7.82	14.51	12.44	9.04	8.15	11.50	10.55	12.49
	TGFB3	1.13	0.77	0.85	0.82	1.54	1.40	0.86	0.48	0.92	2.12	2.56	2.44	1.97	2.12	2.37
	TGFBR1	13.06	9.74	7.26	13.06	11.45	6.89	7.72	6.85	6.66	4.34	3.68	3.47	4.98	4.01	9.11
	TGFBR2	67.74	58.49	40.97	36.64	40.85	22.68	54.71	47.30	58.70	22.79	19.82	19.33	25.13	23.80	30.46
	TGFBR3	6.51	5.72	8.25	23.15	24.00	23.94	2.78	1.97	4.15	7.97	4.97	3.90	5.20	5.38	7.72
	SMAD2	43.40	38.97	32.07	30.97	32.82	24.44	37.58	29.30	34.73	29.10	25.02	24.11	25.99	28.28	32.84
	SMAD3	42.76	31.23	47.02	51.49	57.60	60.29	28.39	29.99	45.34	31.31	26.83	25.64	30.25	35.31	34.56
Proliferation	PIK3CA	7.34	5.79	4.68	8.31	8.71	4.91	4.90	3.20	5.35	5.34	3.86	3.98	5.08	4.57	7.02
	MAPK1	26.22	25.78	19.81	32.74	30.01	28.51	23.98	25.12	26.41	21.51	18.51	17.60	19.45	20.19	24.47
	RAF1	36.72	33.06	29.64	42.42	48.93	48.80	31.34	30.68	34.87	27.15	26.82	24.50	29.53	28.08	33.68
	SOS1	16.88	11.94	10.20	18.39	22.17	15.05	9.44	8.92	9.19	14.71	9.93	8.00	12.67	11.60	16.98
Oxidative stress	MAOA	33.27	24.51	36.65	31.31	35.06	48.24	14.74	11.49	24.90	16.00	13.34	13.15	12.39	13.31	14.18
	OXR1	25.78	20.96	18.53	29.71	34.62	21.22	16.16	12.75	16.29	26.63	24.20	20.32	27.25	27.58	29.93
	SOD1	154.60	145.94	158.76	340.73	319.43	312.28	161.06	131.22	168.23	197.34	194.78	205.12	235.40	222.53	227.92
	GSR	43.09	35.08	49.00	79.89	92.60	88.88	39.01	30.85	59.32	57.76	50.33	43.75	58.89	61.64	69.71
Ferroptosis	FTH1	14623.40	16848.20	19153.50	40456.90	47064.90	38180.30	15009.90	17242.70	18736.70	7827.62	7919.78	8002.75	8133.46	7604.80	8040.58
	SLC40A1	2.51	2.84	2.24	6.37	11.78	4.39	1.45	1.30	3.06	0.75	0.63	0.80	0.52	0.63	0.69
	NFE2L2	71.11	55.31	57.91	90.52	82.32	64.03	41.35	32.54	47.25	68.74	57.30	55.92	65.38	62.17	81.07
	HMOX1	1.78	2.68	2.47	11.93	16.29	15.77	4.49	6.84	5.43	0.93	1.31	1.43	1.04	1.03	0.67
metabolism	HIF1A	68.02	46.88	40.11	67.04	66.51	56.73	47.53	30.90	37.16	108.32	61.83	66.41	81.04	78.77	107.22
	ATP6V1A	22.28	18.11	14.55	27.44	28.29	29.56	12.62	11.37	13.80	16.35	13.15	12.36	17.40	15.69	21.10
DNA repair	OGG1	7.90	9.05	11.28	2.66	2.13	2.38	8.18	7.37	9.44	8.93	11.64	7.91	8.82	9.20	9.63
	XPA	9.40	5.71	4.59	1.32	1.85	2.13	5.98	4.13	5.25	7.21	7.61	9.60	8.80	8.34	7.79
	BRCA1	9.93	7.00	5.76	1.58	1.57	1.47	6.56	4.01	5.17	10.02	7.16	8.52	9.74	8.43	10.70
	BRCA2	4.68	2.47	2.48	0.85	0.43	0.82	4.34	1.92	3.72	4.06	3.39	3.38	4.06	3.02	4.18

Supplementary Figure S1. RNA-seq-based tumor proliferation-associated gene expression (unit: TPM) in SCC-4 cells following 2 days of treatment with arecoline or SAB (full version). RNA-seq: RNA sequencing; TPM: transcript per million; SAB: salvianolic acid B; Expt.: experiment.
